# High Energy Conversion Efficiency with 3-D Micro-Patterned Photoanode for Enhancement Diffusivity and Modification of Photon Distribution in Dye-Sensitized Solar Cells

**DOI:** 10.1038/s41598-017-15110-4

**Published:** 2017-11-08

**Authors:** Min Ju Yun, Yeon Hyang Sim, Seung I. Cha, Seon Hee Seo, Dong Y. Lee

**Affiliations:** 1 Nano Hybrid Technology Research Center, Creative and Fundamental Research Division, Korea Electrotechnology Research Institute, Changwon, South Korea; 2 Department of Electro-functionality Materials Engineering, University of Science and Technology, Changwon, South Korea

## Abstract

Dye sensitize solar cells (DSSCs) have been considered as the promising alternatives silicon based solar cell with their characteristics including high efficiency under weak illumination and insensitive power output to incident angle. Therefore, many researches have been studied to improve the energy conversion efficiency of DSSCs. However the efficiency of DSSCs are still trapped at the around 10%. In this study, micro-scale hexagonal shape patterned photoanode have proposed to modify light distribution of photon. In the patterned electrode, the appearance efficiency have been obtained from 7.1% to 7.8% considered active area and the efficiency of 12.7% have been obtained based on the photoanode area. Enhancing diffusion of electrons and modification of photon distribution utilizing the morphology of the electrode are major factors to improving the performance of patterned electrode. Also, finite element method analyses of photon distributions were conducted to estimate morphological effect that influence on the photon distribution and current density. From our proposed study, it is expecting that patterned electrode is one of the solution to overcome the stagnant efficiency and one of the optimized geometry of electrode to modify photon distribution. Process of inter-patterning in photoanode has been minimized.

## Introduction

Dye-sensitized solar cells (DSSCs) are considered a promising alternative to silicon-based solar cells due to characteristics such as high efficiency under weak illumination and a power output that is insensitive to incident angle^1–3^. These characteristics are more important in urban conditions, where there is a limited time for direct incidence of solar illumination; improvement of the energy conversion efficiency of DSSCs to maintain high performance under weak illumination has the potential to widen the application of photovoltaics in various fields.

A number of studies have focused on improving the peak efficiency of DSSCs using a range of approaches, including modification of organic dyes^4–6^, light trapping^7–10^, modification of the electrolyte^11^ and counter electrode^12^, and enhancement of photoanodes^13,14^, but the efficiency of DSSCs has stagnated at around 10% over the past decade. Therefore, overcoming these limitations requires in-depth re-investigation of the structure of DSSCs, and optimizing some features that are often taken for granted.

One of the special features of DSSCs is their photoanodes. Unlike other kinds of solar cells, including silicon- based solar cells, thin-film solar cells, and organic solar cells, DSSCs have thick light-absorbing electrodes, i.e., photoanodes, of 10~20 *μm*, almost 100 times thicker than other solar cells. Therefore, the generation of electrons in the dye and diffusion and recombination of electrons through the electrode occur simultaneously according to the continuity equation^15^:

1 $$-\nabla \cdot ({\rm{D}}\nabla n)-k(n-{n}_{0})=\lambda I(x)$$

where *D* is the diffusivity of the electrons in the electrode, *k* is the recombination rate, λ is the absorption coefficient of the photoanode, *I*(*x*) is the photon intensity distribution within the photoanode, and *n* is the electron concentration. According to this equation, the generated electrons can be gathered rapidly by increasing the electron diffusivity. Additionally, the distribution of *I*(*x*) determines the electron generation location. A number of previous studies have reported increasing the diffusivity of electrons by utilizing nano-wires or nano-tubes instead of Titanium Dioxide (TiO_2_) nanoparticles^16–22^. Several other studies have focused on enhancing *I*(*x*) by including a scattering layer^23^, or using of photonic crystals^24^ or stamped patterns on the top of the electrode^25–35^. However, the dominant photoanode design has been flat photoanodes with scattering layers, where *D* can be only modified using material modification and *I*(*x*) is determined by the addition of the Beer-Lambert law distribution and scattered light.

However, considering the characteristic feature of DSSCs photoanodes, i.e., the thick light-absorption electrode, another method for enhancing DSSCs performance involves modification of the microscopic morphology of the photoanode. Recent studies have shown that splitting of photoanodes into several parts increases the diffusivity of electrons^36,37^, and the photoanode thickness is sufficient to modify the light distribution of the photon inside by changing the electrode shape. Therefore, it is useful to verify how escaping from the typical design of uniform thick layered photoanode affects DSSCs performance.

In this study, a micro-scale patterned photoanode is proposed, with a hexagonal shape to fill the area compactly within the limited active area utilizing a conventional screen printing method. The patterned photoanode can be fabricated simply and easily by a one-step process and formed without overlap between the patterns. To analyze the effect of the patterned photoanode, the photovoltaic performance and diffusivity behavior was investigated for different thicknesses and various diameters of the pattern. Additionally, the light behavior of the inner photoanode, which affects the charge generation and transport and drives high energy conversion efficiency, was simulated by controlling the diffusivity coefficient and current in the continuity equation for different photoanode thicknesses and morphology.

## Results and Discussion

**Figure 1:**
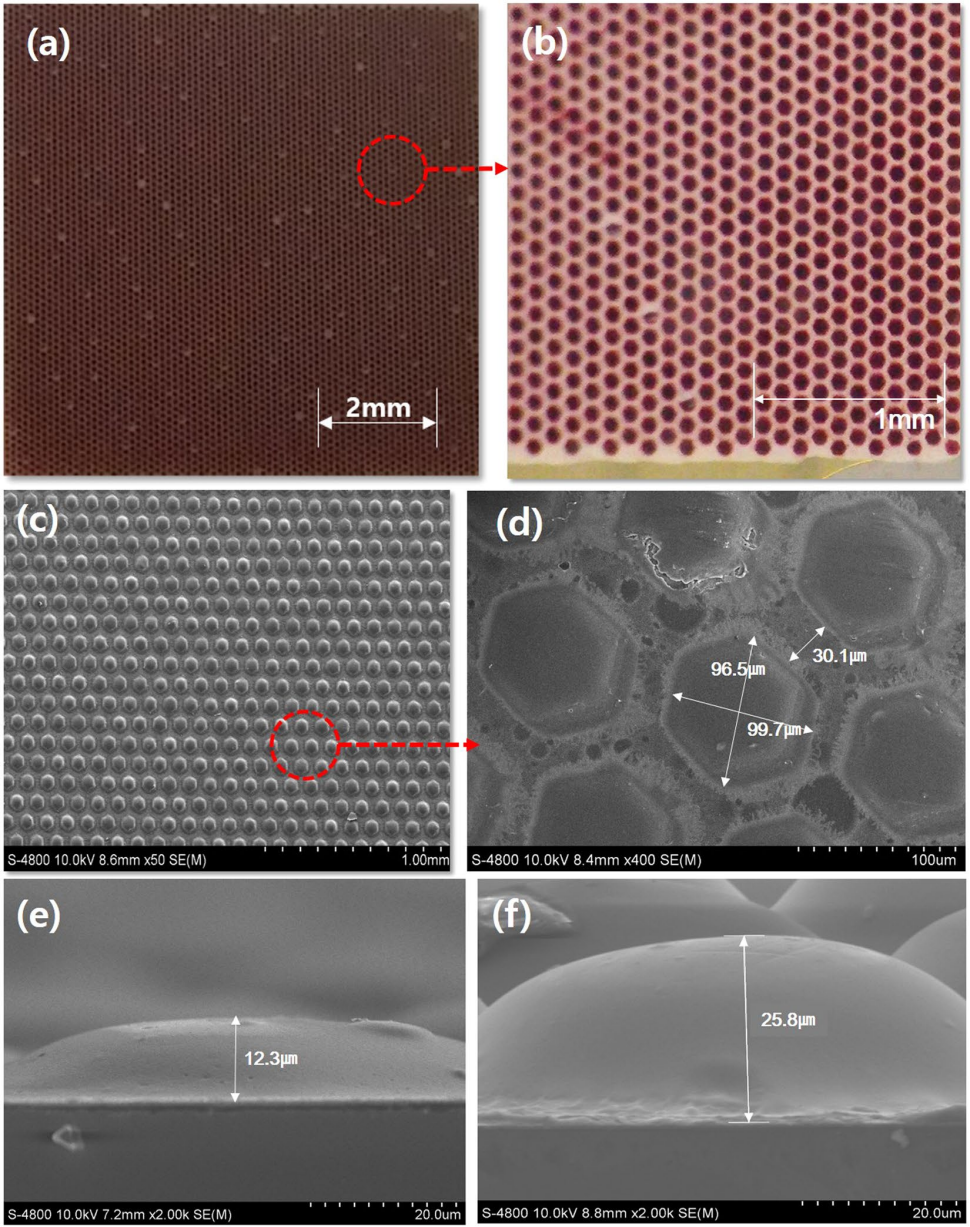
Optical and scanning electron microscope (SEM) micrographs of patterned photoanode of DSSCs of 100 *μm*pattern size: (**a**) low-magnification and (**b**) high-magnification optical images of patterned photoanode; (**c**) SEM micrographs of hexagonal array of patterns deposited by repeated screen-printing followed by sintering and dye staining and (**d**) magnified view showing their dimensions; (**e**) and (**f**) show cross-sectional images of patterns of different thicknesses controlled by a number of repeated screen-printing processes.

The patterned photoanodes were prepared by repeated screen-printing with a patterned screen-printing mask, followed by sintering and dye staining. The patterns had a hexagonal shape in-plane and were arrayed to fill the area without loss. The patterns ranged from 100 *μm* to 1000 *μm* with the spacing between patterns maintained at 30 *μm*, except for the 1000 *μm* pattern, where the spacing was 50 *μm* to avoid pattern overlap during the repeated screen printing process (Fig. S1). Figure 1(a) presents an example of the patterned electrodes for the 100 *μm* pattern size. The pattern cannot be seen at the sample scale, but the hexagonal array can be seen in the magnified image, as shown in Fig. 1(b) and (c). The more detailed view reveals that each pattern has a hexagonal shape, as designed. The figures show that the spacing between patterns is uniform and is actually empty, so photons incident in this area cannot by converted to electricity and be lost. Thus, if the spacing between patterns is decreased then the loss can also be decreased. The cross-sectional view of these patterns, shown in Fig. 1(e) and (f), reveals that the electrode consists of a large number of semi-spherical domes rather than vertical pillars. This characteristic cross-sectional shape may be induced by the surface tension of TiO_2_ paste during the screen-printing process. One interesting feature of the cross-sectional morphology of the pattern is that the shape is maintained even though the thickness is increased by repeated deposition, as shown in Fig. 1(e) and (f).

**Figure 2:**
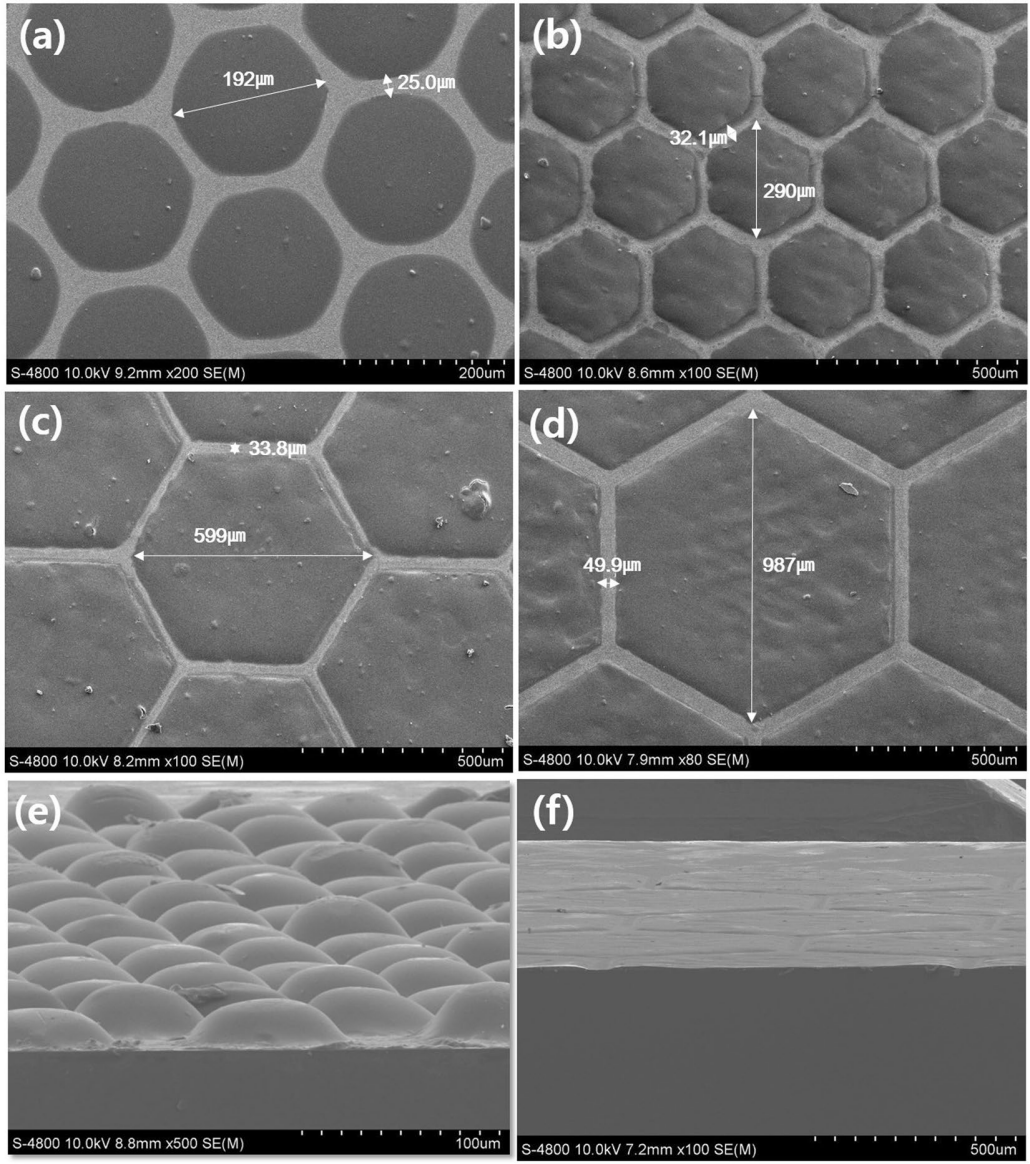
SEM micrographs of patterned photoanodes of various pattern sizes: Plane view of patterned photoanode of (**a**) 200 *μm* pattern size, (**b**) 300 *μm* pattern size, (**c**) 600 *μm* pattern size and (**d**) 1000-*μm* pattern size; (**e**) and (**f**) show cross-sectional SEM images of a patterned photoanode with a 100 *μm* pattern size and a 1000 *μm* pattern size, respectively.

The patterned electrodes were also deposited with fine geometry as shown in Fig. 2(a–d). The area loss decreased with increasing pattern size and decreasing pattern spacing. One interesting feature in the patterned electrodes is that the cross-sectional morphology of the photoanodes is dependent on the size of the patterns, as shown in Figs 1e and 2e,f. For the small pattern, the cross-sectional morphology maintains a dome shape, but with increasing pattern size, the upper region becomes flat and the edge of pattern becomes thicker, and as a result the cross-sectional morphology of the 1000 *μm* patterned electrode is hat-shaped with a thicker edge region compared to the central flat region (Fig. S2). These cross-sectional morphologies can be explained by the combination of the surface tension of the TiO_2_ paste and contact with the screen-printing masks. However, a detailed shape-forming mechanism should be developed, and future research should yield control methods for cross-sectional morphology.

**Figure 3:**
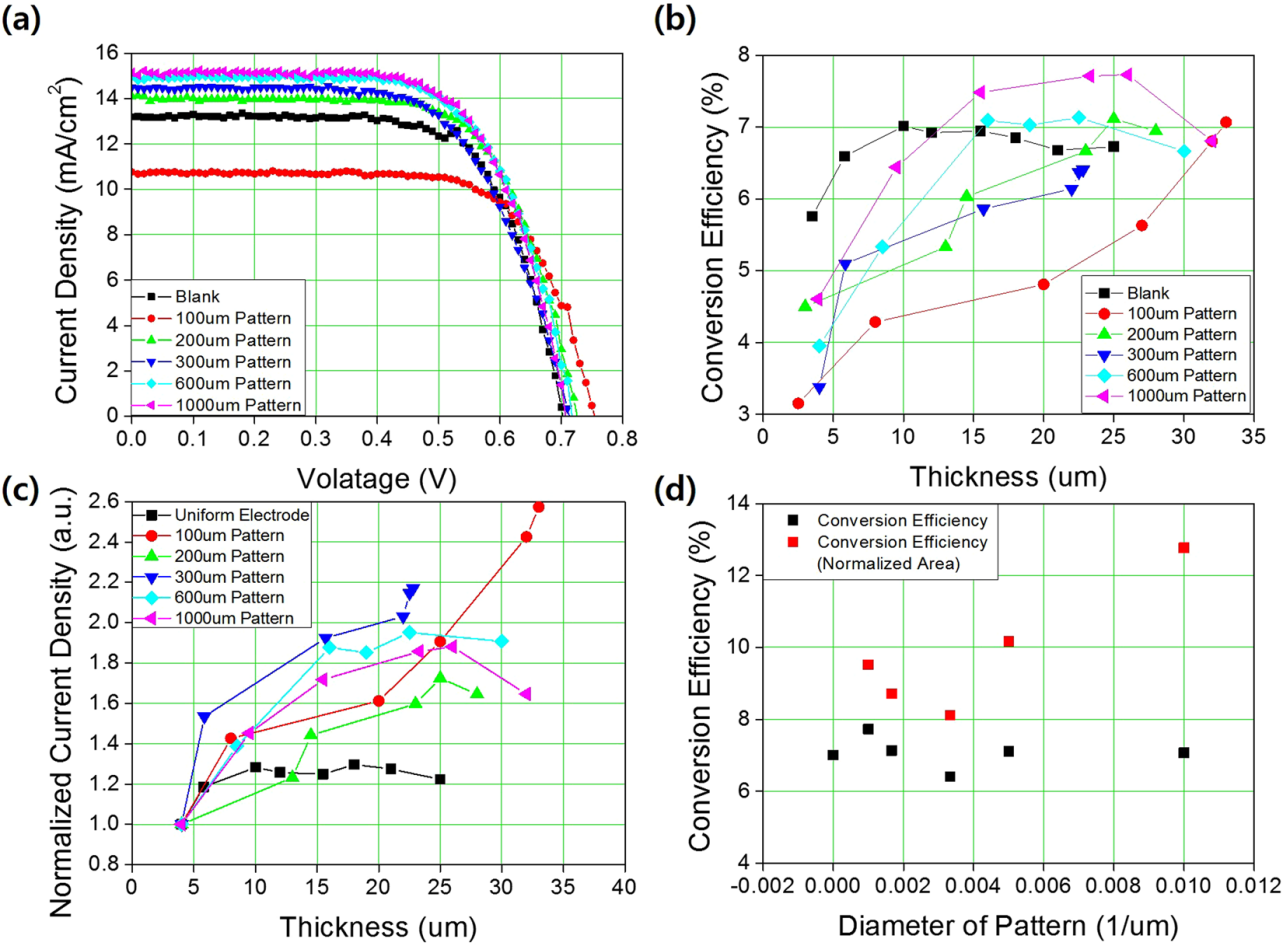
(**a**) Relationship between current density and applied voltage (I-V curve) of uniform and patterned DSSCs with various pattern sizes. (**b**) Variation of power conversion efficiency of uniform and patterned DSSCs of different pattern sizes according to photoanode thicknesses. (**c**) Normalized short circuit current density of uniform and patterned DSSCs with different pattern sizes according to the thickness of the photoanode. These are normalized by the short circuit current density of the smallest value of each condition to show the effect of electrode thickness. (**d**) The power conversion efficiency (black squares) and photoanode area normalized efficiency (red squares) of DSSCs with uniform and patterned photoanodes, according to pattern size.

Considering the area loss due to the pattern spacing, the power conversion efficiency of the assembled DSSCs (Fig. S3) under 1 sun, 1.5 AM illumination was not as expected, as shown in Fig. 3. In the relationship between the current density and the applied voltage (I-V curves) shown in Fig. 3(a), measured from various pattern sizes with similar thicknesses of 20~25 *μm*, as shown in Fig. S4, the current density or efficiency of the patterned electrode of sizes 200, 600, and 1000 *μm* were higher than those of the uniform electrode, and the photovoltaic performances are listed in Table 1. These tendencies can be clearly shown in the relationship between the power conversion efficiency and the thickness of the electrode in Fig. 3(b) (or, in the case of the patterned electrode, the maximum height in the cross-sectional view, as shown in Fig. S5). In the uniform electrode, the efficiency or current density increases with increasing thickness until the thickness reaches 15 *μm* and then decreases with a further increase in thickness, hence showing an optimized thickness of around 15 *μm*, which agrees well with previous studies. One interesting feature of the patterned electrode is that the optimum thickness increased with decreasing pattern size. For a pattern size between 600~1000 *μm*, the optimum thickness ranged from 20~25 *μm*, but for a pattern size of less than 300 *μm*, the efficiency increased with increasing thickness until thicknesses greater than 25 *μm*. See Supporting Information Fig. S6 for more detail values of each condition. The effect can be seen more clearly when the short circuit current density (*J*_*sc*_) measurements are normalized to their minimum value, i.e., *J*_sc_ at the minimum thickness. Figure 3(c) shows the relationship between these normalized values and the electrode thickness: the optimum electrode thickness clearly increases for patterned electrodes, and increases with decreasing pattern size.

**Table 1:** Performance of uniform and patterned DSSCs with various pattern sizes.

Pattern Size (*μm*)	J_sc_ (*mA/cm* ^2^)	V_oc_ (*V*)	Fill Factor	Efficiency (%)	Thickness (*μm*)
*Uniform*	13.21	0.7	0.72	6.66	25.0
*100*	10.76	0.75	0.7	5.6	25.0
*200*	14.08	0.72	0.68	6.95	25.0
*300*	14.44	0.71	0.68	6.64	22.8
*600*	14.80	0.72	0.66	7.12	22.5
*1000*	15.03	0.73	0.72	7.73	26.0

The values are averages measured from samples from the same processing route.

Another interesting feature is that the maximum efficiency of the patterned electrode is higher than that of the uniform electrode. In the case of the uniform electrode, the maximum efficiency among the tested DSSCs is around 7.1%, but this increased to 7.8% when the pattern size was 1000 *μm*, as shown in Fig. 3(d), and the maximum efficiencies of patterned electrodes exceeded that of the uniform electrode except for the pattern size of 300 *μm*. Efficiency was calculated based on the deposited area, within which the spacing between patterns is included. Hence, the patterned electrode has an area loss due to the spacing between patterns, which is fixed at 30 *μm* or 50 *μm*, and the actual photoanode area is 55% of the deposited area when the pattern size is 100 *μm* and 81% when the pattern size is 1000 *μm*. See Supporting Information Table S1 for more details. This area fraction can be increased by decreasing the spacing between patterns using a more precise screen-printing process, so these results indicate that the patterned electrodes are much more efficient than the uniform electrode. To clarify this result, the actual photoanode area where the photon is incident was considered by excluding the spacing between the patterns, as shown in Fig. 3(d). Here, the patterned electrode has an improved performance and the photoanode area-based efficiency reaches 12.8% when the pattern size is 100 *μm*.

**Figure 4:**
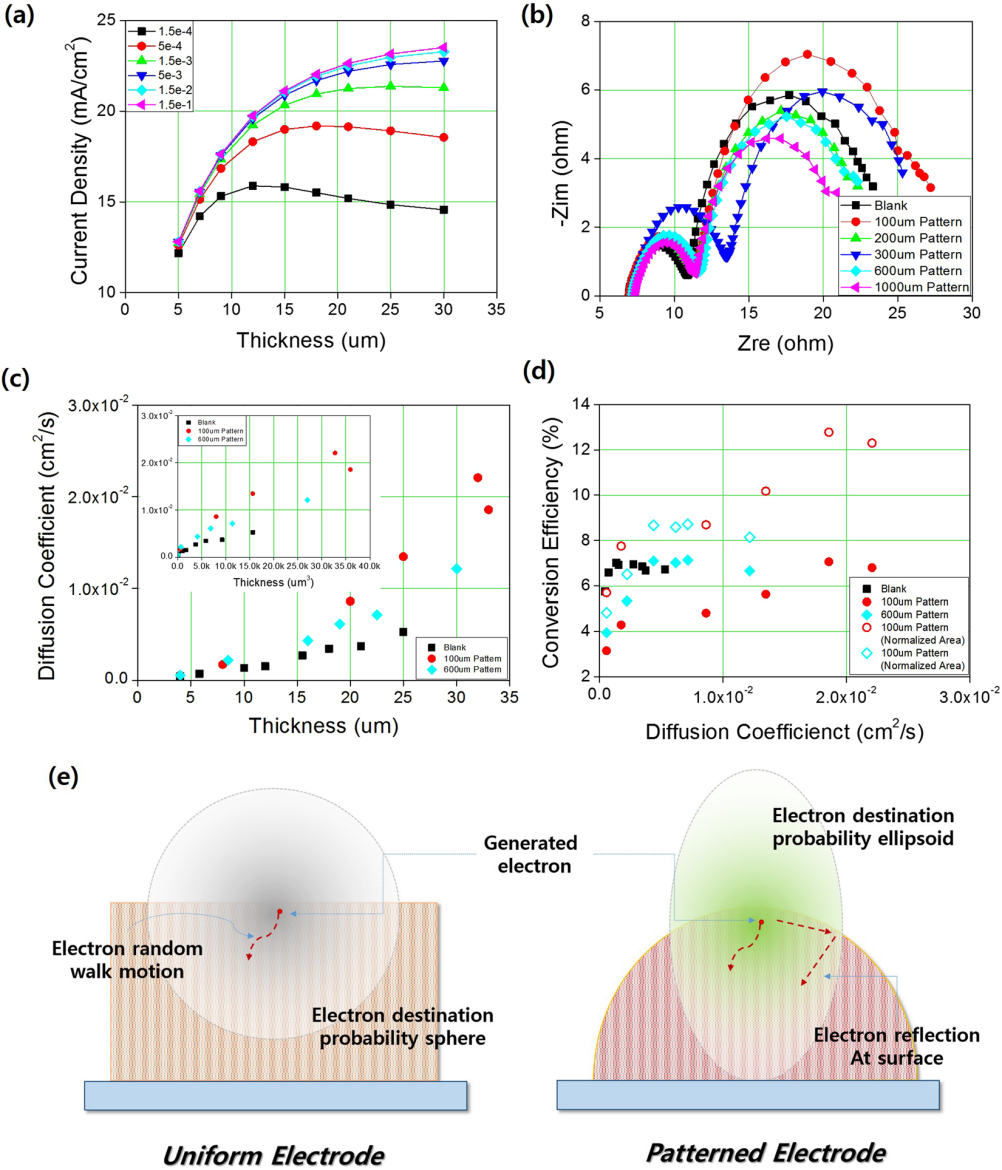
(**a**) FEM results showing the effect of diffusivity and thickness of uniform photoanode on the short circuit current density of DSSCs. (**b**) EIS Nyquist plot of DSSCs with uniform and patterned photoanode of different pattern sizes measured under 0 V, 1 sun conditions. (**c**) Electron diffusivity of each DSSCs with uniform and patterned photoanodes according to the thickness of electrodes and (inset) according to the cube of the thickness. (**d**) Graph showing the effect of electron diffusivity on the efficiency (filled marks) and the photoanode area normalized efficiency (open marks). (**e**) Schematics illustrating the effect of pattern size on the electron diffusivity in uniform (left) and patterned (right) DSSCs photoanodes.

Considering that the only modification is the morphological change from a uniform electrode to a patterned electrode, and given that the same materials were used in each case, including TiO_2_ nanoparticles, dye, electrolyte, and counter electrodes, it is unlikely that the performance improvements arose from different recombination rates, expressed as *k* in eq. (1). Instead, the morphology changes might induce modifications in diffusivity and photon distribution within the electrode. For diffusivity, the relationship between the thickness and the efficiency or short circuit current shown in Fig. 3(b) and (c) resembles the relationships between these parameters at different diffusivities in the uniform electrode, calculated using the finite element method and shown in Fig. 4(a). In this relationship, for fixed diffusivity, *J*_sc_ increased up to the optimum thickness and decreased in the thicker electrodes. When the diffusivity increased, the optimum thickness increased so that electrons generated from the thicker electrode region could be gathered into *J*_sc_ and avoid recombination. Also the relationship the amount of dye absorption and the conversion efficiency as shown in Fig. S7 have analyzed to consider the portion of the electron generation. The patterned electrode exhibits high conversion efficiency with smaller amount of dye absorption in other words, smaller amount of the generated electron concentration, which is the evidence that the light is highly utilizable and diffusion is much faster within the patterned electrode compare to the uniform electrode. To verify the effect of morphology on performance, the diffusivity of the uniform and patterned electrodes were measured and calculated from the electrochemical impedance spectroscopy (EIS) results shown in Fig. 4(b), and an additional Bode plot is shown in Fig. S8 ^38^. As shown in Fig. 4(c), the diffusivity of the patterned electrodes was much larger than that of the uniform electrodes, and a smaller pattern induced a higher diffusivity. For the 100-*μm* pattern size, the electron diffusivity was almost 10 times higher than that of the uniform electrode; See Supporting Information Fig. S9 for the diffusivity of each patterned electrode and uniform electrode with increasing thickness. The mechanism for the improved diffusivity in the patterned electrodes according to pattern size is not clear at this stage and needs more study. However, the relationship between thickness and diffusivity shown in Fig. 4(c) provides some indications. In the case of the uniform electrode, the diffusivity has little dependence on the thickness of the electrodes, but in the case of the patterned electrodes, the diffusivity increased with increasing thickness and the logarithm of the diffusivities had a linear relationship to the cube of the thickness, i.e., log *D* is proportional to *d* ^3^ where *D* is diffusivity and *d* is thickness. This implies that the diffusivity is related to the volume of the electrode, where the electrons are diffused to be gathered into the current. If electrons move in a random walk, the electron destination is likely to form a sphere if no field or chemical potential is applied, as shown in Fig. 4(e), and the radius of the sphere is proportional to the mean free path of the electron. However, if the electrodes are patterned, diffusion in the lateral direction is restricted by geometrical confinement and should be reflected from the surface, and the spherical probability distribution could be modified to an ellipsoidal shape in the direction of thickness. However, with regard to the relationship between the diffusivity and the efficiency of the patterned and uniform electrodes, shown in Fig. 4(d), the diffusion effect does not provide a complete explanation for the enhanced efficiency.

Along with electron diffusion, the photon distributions in the electrodes are expected to play an important role in the performance enhancement of patterned electrodes. As shown in the micrographs of patterned electrodes in Figs 1 and 2, the electrode morphologies are dependent on the pattern sizes. In the case of the uniform electrode, the photon distribution is expected to have a 1-dimensional Beer-Lambert law relationship, but the patterned electrodes have a more complex photon distribution because the scattered and reflected photons on the surface of the electrode may influence the photon distribution within the electrodes. To estimate the morphological effect on the photon distribution and resulting current density, finite element method analyses of photon distributions were conducted using the Helmholtz equation, which treats light as a wave as in eq. (2), where *u* is the wave function, *k* is the wave number, and *f* is the source function of light:

2 $${\nabla }^{2}u+{k}^{2}u=f$$

**Figure 5:**
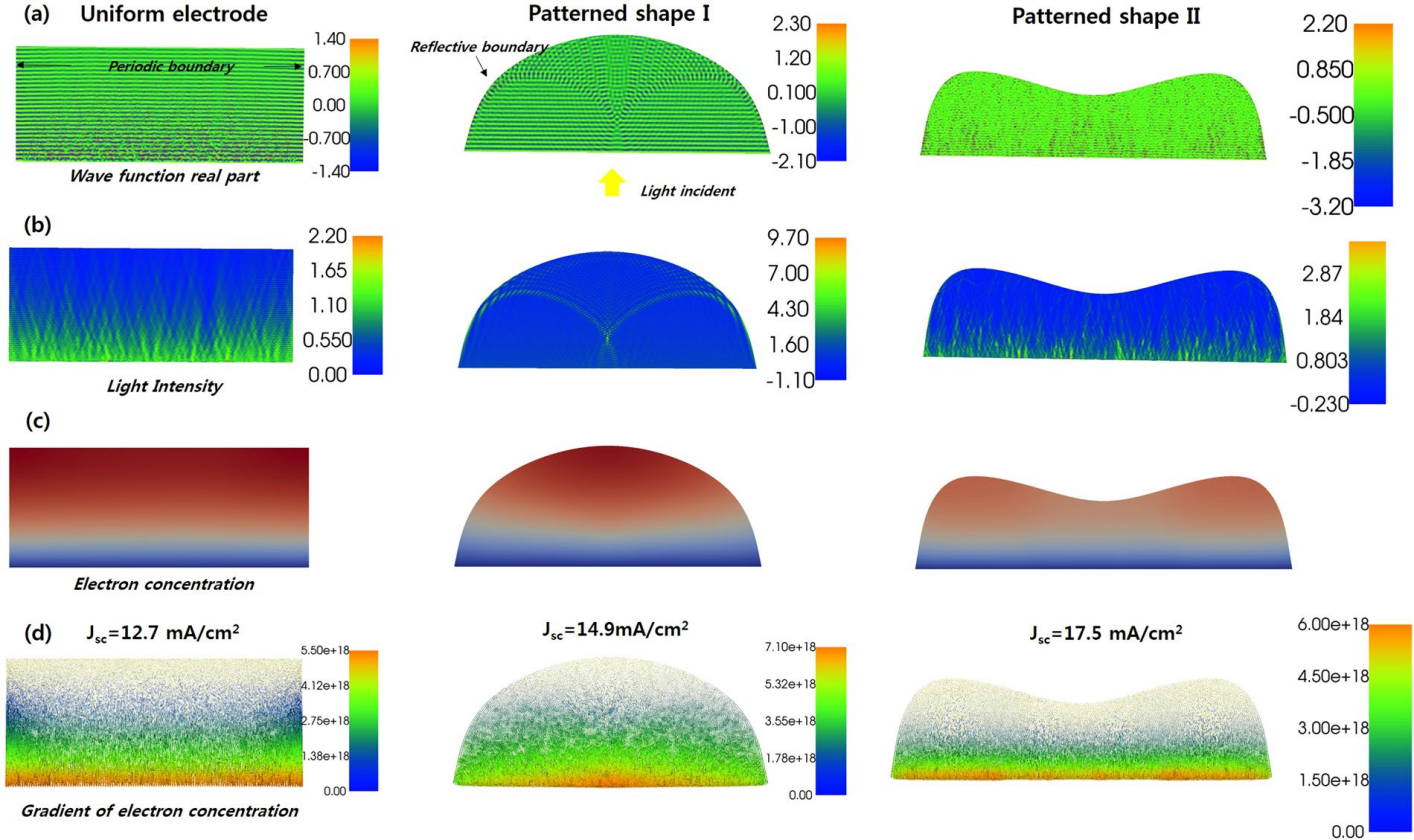
FEM results of uniform shaped photoanode (left), dome shaped patterned photoanode (middle), and saddle shaped patterned photoanode (right) of DSSCs under illumination of unit intensity of 550 nm monochromatic incident light. The thickness of the electrode was 8 *μm* and the width of the pattern was set at 20 *μm*. (**a**) Real part of the complex wave function within the electrode. (**b**) Light intensity and (**c**) concentration of electrons generated by light distribution calculated by (**b**). (**d**) Gradient of electron concentration within the electrodes, indicating the internal current (within the electrode) and current density of DSSCs (lower boundary).

In addition to the light distribution, the resulting photon intensity was applied to the electron contiguity equation expressed in eq. (1) to obtain the effect of the photon distribution on *J*_sc_ shown in Fig. 5. In the calculation, the light was illuminated with unit intensity to the lower part dominant in Fig. 5 and the other boundary was set as a Neumann boundary condition to estimate the reflection of light at the interface of the photoanode and the scattering layer. In this case, the gradient of the wave function was zero on the interface, which implies that half of the light is reflected at the surface (see Supporting Information for more detailed calculation procedures.) The real part of the complex wave function, shown in Fig. 5(a), which indicates the propagation of the optical wave, shows that the patterned electrode has more complex wave propagation behavior according to shape, whereas the uniform electrode has 1-dimensional wave propagation. The complex wave propagation behavior induced local light concentrations such as in lenses and mirrors within the electrodes, as shown in Fig. 5(b). One interesting feature is that in the pattern shape shown in the small pattern size, indicated as pattern shape I, the light concentration occurs near the surface, while in pattern shape II it occurs near the interface between the electrode and Fluorine-doped Tin Dioxide (FTO), where electrons are gathered. The photon distribution within the electrode affects the distribution of generated electrons, as shown in Fig. 5(c), and consequently affects *J*_*sc*_, which is determined by the gradient of the electron concentration within the electrodes, as shown in Fig. 5(d). In this calculation, electrode shape II has the maximum current density for the same thickness and diffusivity. However, it should be noted that these results are from a single wavelength of 550 nm and under highly ideal conditions. Additional considering the continuity equation, the exciting and guiding path of electrons can be regarded as light path and diffusivity. When the incident light passes through the photoanode which have the geometric characteristic, the electron flux is formed from the light distribution as shown in Fig. 5 (b) and (c) and then the diffusion path is formed from the guiding of the electron flux. Light distribution and diffusion both factors have significant impact on the photovoltaic performance, so in the patterned electrode, impact of the geometric characteristic to diffusion is enhanced by more electron flux increase the J_sc_ and conversion efficiency. It is well matched with the assumption of the electron path within the photoanode as shown in Fig. 4 (e). For more detailed and accurate results, the effect of scattering layer should be treated more precisely, instead of using just simple reflection and the calculation should be expanded to the whole wavelength range of solar illumination. Despite this limitation, these results indicate that the shape of the electrode has a significant effect on light distribution within the electrode and hence the performance of DSSCs. The photon distribution within the electrode is very sensitive to the shape of electrodes, so further research is required to identify an optimized morphology for DSSCs photoanodes. This has not yet been considered in depth, but may be a key factor in overcoming current efficiency limits. In particular, considering the enhanced diffusivity from geometric restriction, the patterned electrode provides a good starting point for a break-through in DSSCs efficiency.

## Summary

Despite the many characteristic advantages of DSSCs, including high efficiency under illumination and insensitivity to incident angle of light, their efficiency has stagnated around 10%, limiting their widespread use in photovoltaic applications. To overcome this limitation, utilizing the characteristic feature of DSSCs photoanodes including their thick light absorption layer, this study developed patterned electrodes fabricated using a repeated screen-printing process. The pattered electrode has hexagonal shape and it were arrayed compactly with minimizing the loss. The patterned electrode increased efficiency, based on the power produced per deposited area, from 7.1% to 7.8%. When the efficiency was calculated based on the photoanode area, an efficiency of 12.7% was obtained. The enhanced performance of these patterned electrodes arose from two major factors: the enhanced diffusion of electrons within the electrode by geometrical restrictions and modification of the photon distribution within the electrode. From the finite element method analyses results, according to the morphology of electrode, the light distribution affects the distribution of generated electrons which is determine the gradient of the electron concentration that is related to the J_sc_. Therefore, patterned electrodes provide a good starting point for overcoming the stagnant efficiency of DSSCs via more in-depth investigation of the diffusion behavior of electrons, geometrical restriction, and optimization of electrode geometry to modify photon distributions. This process should be improved to minimize the inter-pattern spacing, which induces area loss in electrodes.

### Experimental Details

Fluorine-doped tin oxide (FTO, sheet resistance 7 Ω sq^−1^, Sigma Aldrich) glass was used as the substrate for the photoanode and the counter electrode. The FTO glass was rinsed with acetone, ethanol, and distilled and deionized water by sonication for 30 minutes and dried with nitrogen gas. A blocking layer (Solaronix) was deposited on FTO glass by automatic screen printing (AutoMax) then heat-treated at 530 °C for 3 h in air. On the blocking layer, 20-nm TiO_2_ nanoparticles (Solaronix) were deposited over an area of 8 mm × 8 mm for both the patterned photoanode and the uniform photoanode, and then heat treated at 500 °C for 1 h in air. Finally, 500 nmTiO_2_ nanoparticles (ENB Korea) were deposited as the scattering layer, and then heat treated at 500 °C for 1 h in air. The TiO_2_ deposited FTO glass was immersed in a 0.3 mM ethanol solution (Sigma Aldrich) of N719 dye (Sigma Aldrich) at room temperature for 20 h. For the counter electrode, platinum paste was deposited on FTO glass masked using 3 M tape by a doctor blade, and then heat-treated at 450 °C for 30 m in air. The TiO_2_ photoanode was assembled with the counter electrode using surlyn film (60 *μm*. Solaronix) and liquid electrolyte was injected through the hole on the FTO glass of the counter electrode. The liquid electrolyte was composed of 0.6 M 1-butyl-3-methylimidazoli*μm* iodide (BMImI), 0.03 M iodine, 0.1 M guanidine thiocyanate, 0.5 M 4-tert-butylpyridine, and 0.1 M lithium iodide dissolved in mixed Acetonitrile and 3-methoxypropionitrile with 2:8 volume ratio.

Field-emission scanning electron microscopy (FE-SEM, Hitachi S4800) was performed to observe the surface and the cross-section of the patterned photoanode. The photovoltaic performance of the DSSCs was evaluated using a solar simulator (Abet Technologies, model Sun 2000, 1000 W Xe source, Keithley 2400 source meter) under 1.5 AM, 1 sun condition, calibrated by a KG-3 filter and a NREL-certified reference cell. The results of parameters of photovoltaic performance are averaged by measuring three DSSCs in each pattern size condition and a shading mask was applied for clarifying the active area of 0.283 mm^2^. Electrochemical characterizations were performed using a BioLogic SP-300 potentiostat. The impedance spectra were acquired under 0 V, 1 sun condition.

Finite element method calculations were performed using the FEniCs module in the Python programming language. For analysis of the electron continuity equation (Eq. (1)), a 2-dimensional model was constructed and calculated using code described in previous research^39,40^. For the photon distribution within the patterned and uniform electrodes, the same methods were utilized with a model width of 20 *μm*. The calculations were conducted using a weak formulation of the Helmholtz equation and the resulting wave function was used to calculate the photon intensity. The photon distribution was applied to *I*(*x*) term in Eq. (1) to calculate *J*_*sc*_ for the patterned and uniform electrodes (see the Supporting Information for more details, including the weak formulation).

## Electronic supplementary material

Supplementary Information
