# Supplementary material for: High Energy Conversion Efficiency with 3-D Micro-Patterned Photoanode for Enhancement Diffusivity and Modification of Photon Distribution in Dye-Sensitized Solar Cells
Source: Sci Rep. 2017 Nov 8;7:15027. doi: 10.1038/s41598-017-15110-4 (PMC5678131; doi:10.1038/s41598-017-15110-4)
Supplement: Supplementary file 1 — Supplementary Information [file 41598_2017_15110_MOESM1_ESM.pdf]

Supplementary Information

High Energy Conversion Efficiency with 3-D Micro-Patterned Photoanode for Enhancement Diffusivity and Modification of Photon Distribution in Dye-Sensitized Solar Cells

Min Ju Yun[1], Yeon Hyang Sim[1,2], Seung I. Cha*[1,2], Seon Hee Seo[1], Dong Y. Lee[1,2]

1. Nano Hybrid Technology Research Center, Creative and Fundamental Research Division, Korea Electrotechnology Research Institute

2. Department of Electro-functionality Materials Engineering, University of Science and Technology

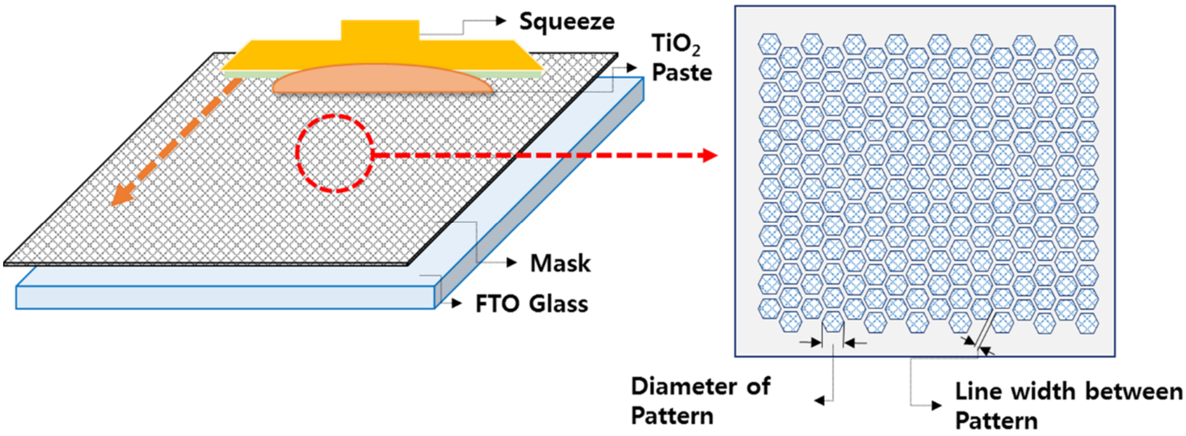

Figure S1. Schematic illustration of screen printing utilizing the mask for depositing patterned TiO_2 paste on FTO glass and right side illustration of mask with hexagonal shape pattern which are arranged compactly in active area.

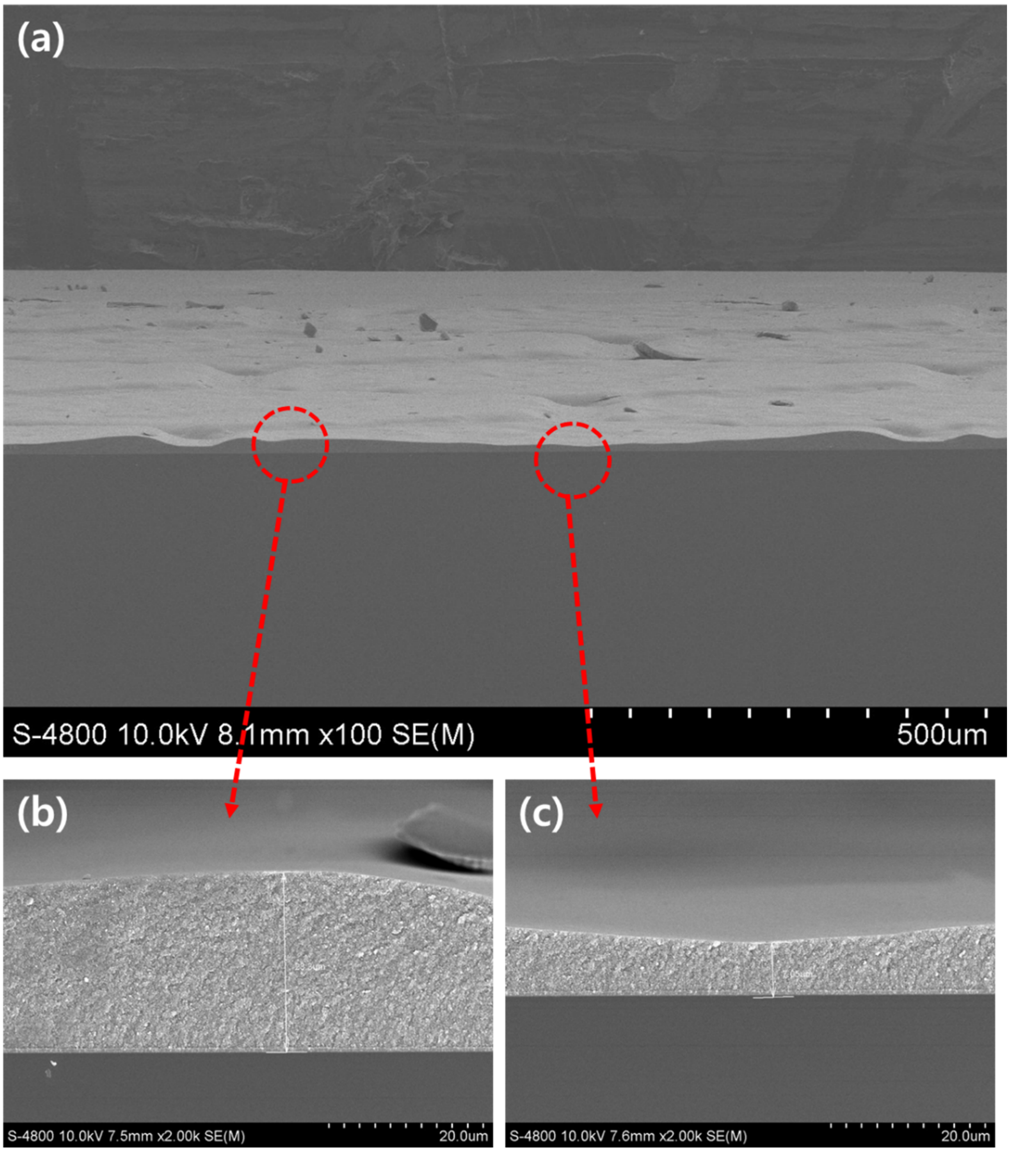

Figure S2. Cross sectional SEM micrographs of (a) 1000 ㎛ pattern size, (b) thick portion and (c) thin portion of it.

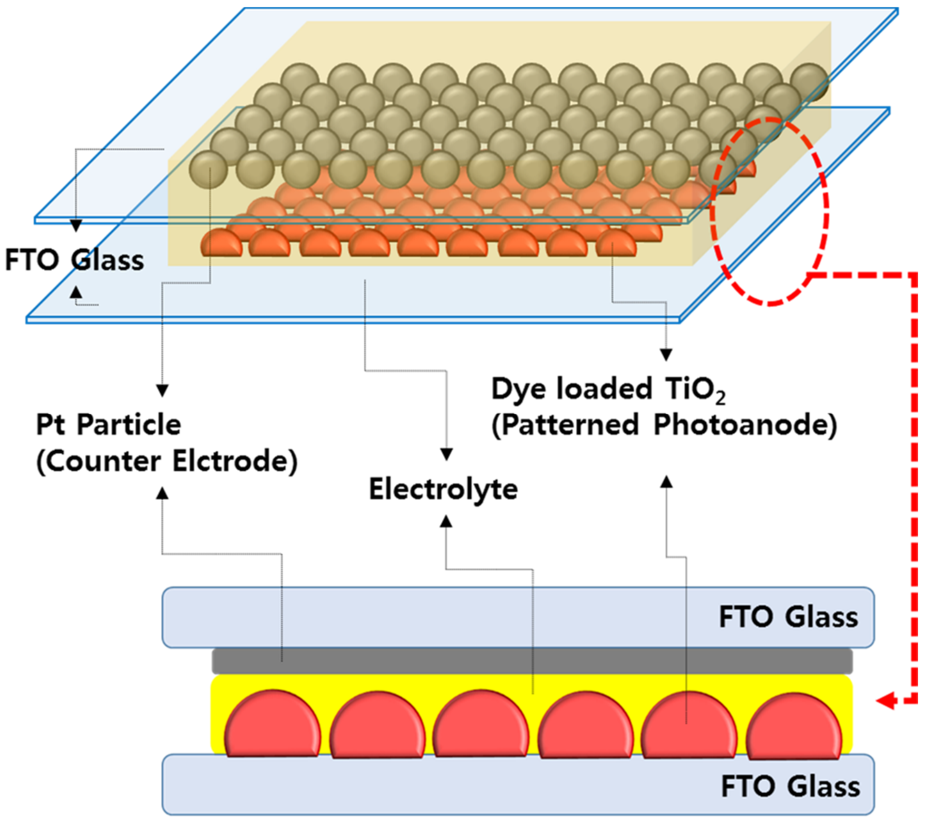

Figure S3. Schematic illustration of assembling process with prepared photoanode and counter electrode. Bottom illustration of schematic cross sectional view of assemble cell.

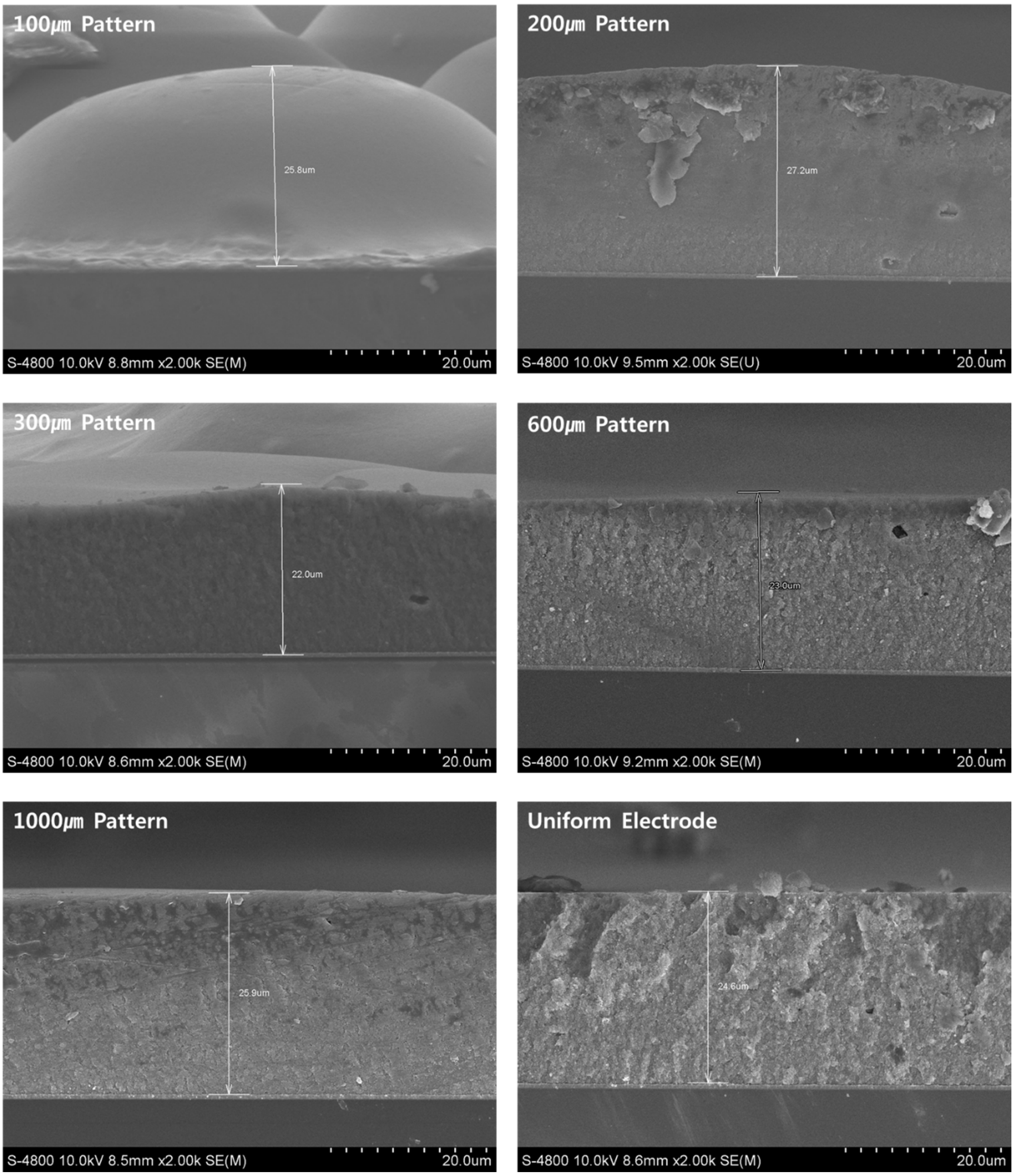

Figure S4. Cross sectional SEM micrographs of patterned electrodes and uniform electrode with similar thickness of 20~25 ㎛.

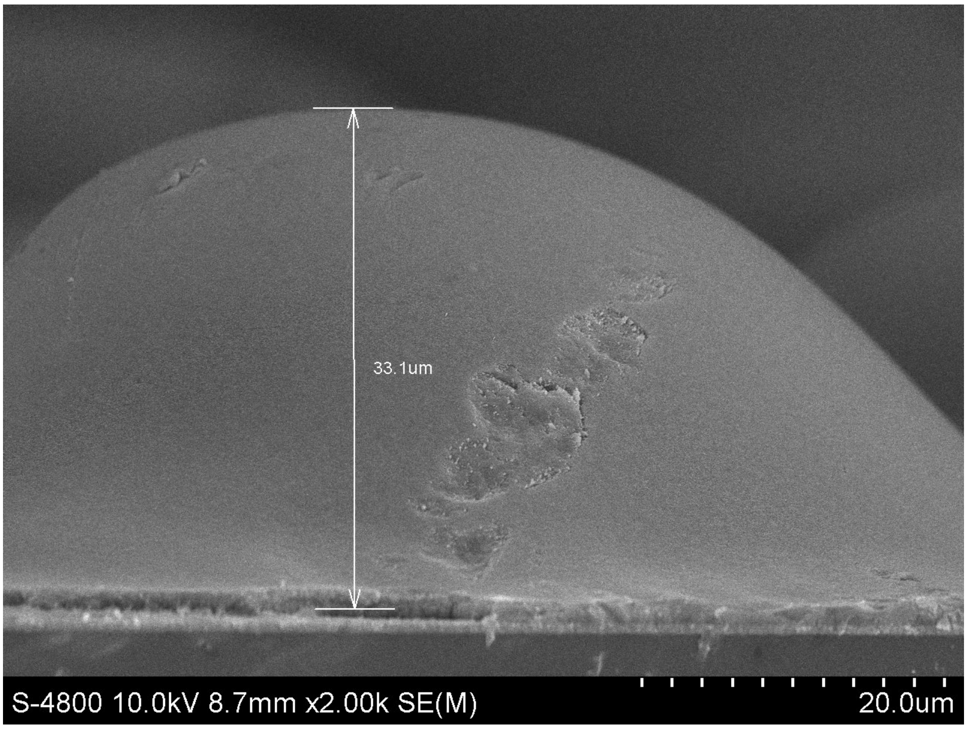

Figure S5. Cross sectional SEM micrographs of maximum height 33.1 ㎛ of 100 ㎛ pattern size.

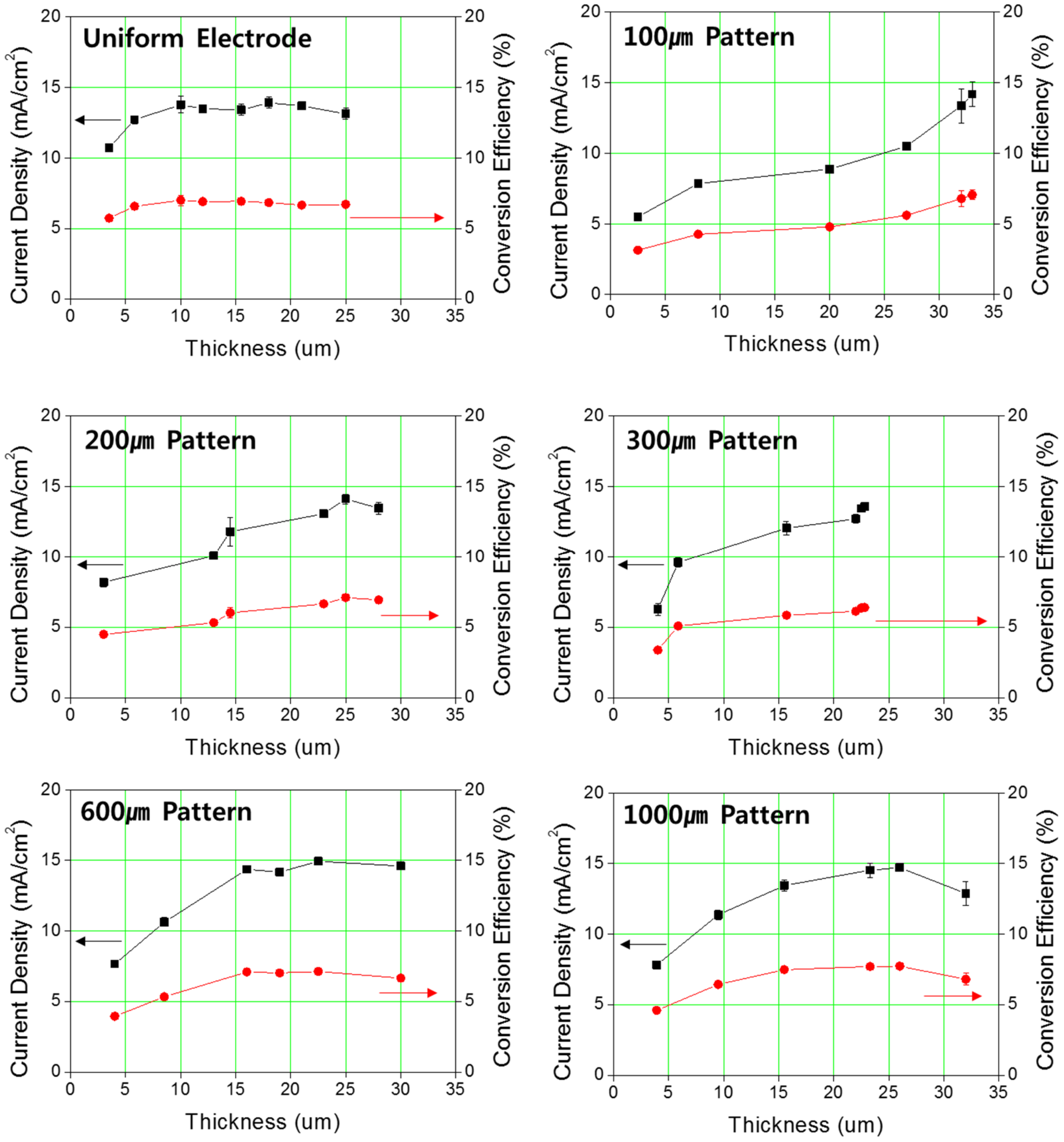

Figure S6. Relationship of current density and conversion efficiency with increasing thickness of uniform and patterned DSSCs with various pattern size.

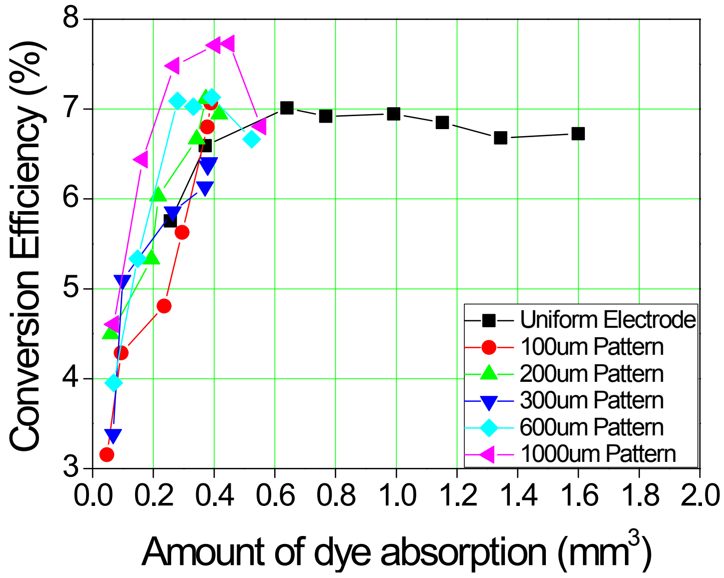

Figure S7. Relationship of the amount of dye absorption and the conversion efficiency of uniform and patterned DSSCs with various pattern size.

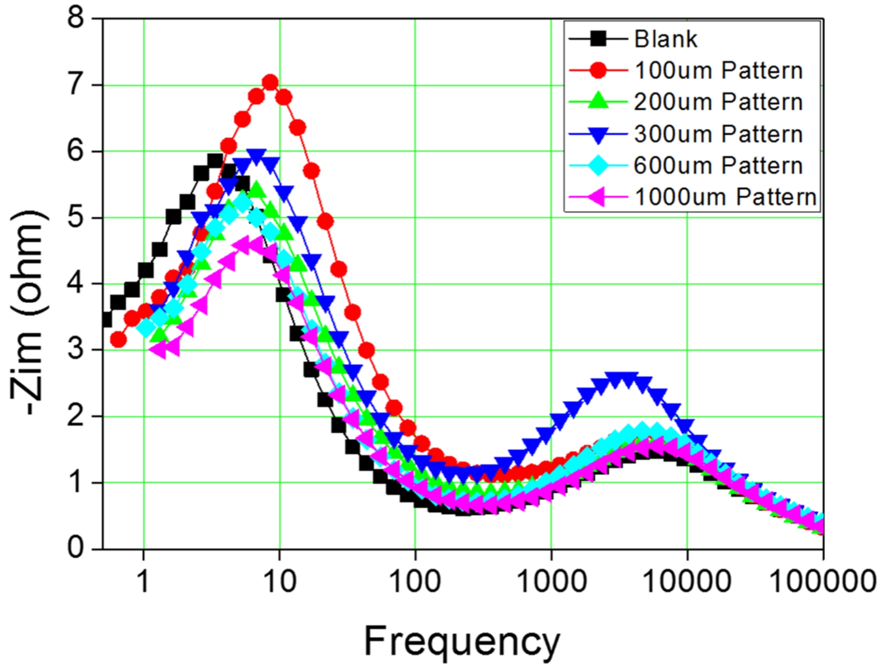

Figure S8. EIS Bode plot for uniform and patterned DSSCs under 0V, 1sun condition according to pattern size.

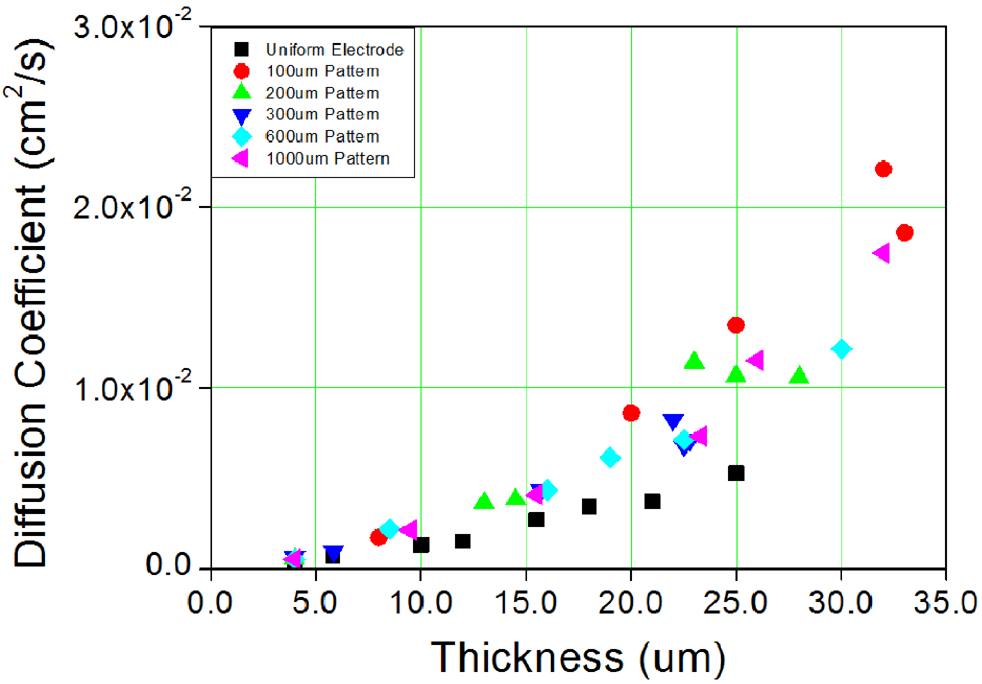

Figure S9. Diffusivity of uniform and various patterned DSSCs with increasing thickness of photoanode.

Table S1. Actual photoanode area and deposited photoanode area of uniform and various patterned electrode. Actual photoanode area is calculated excluding spacing area between patterns.

**Pattern Size (㎛)**	**Actual Photoanode Area (*mm2*)**	**Deposited Photoanode Area (*mm2*)**
Uniform	64.00	64.00
100	35.40	64.00
200	44.75	64.00
300	50.51	64.00
600	52.38	64.00
1000	51.96	64.00

Finite Element Analysis of Light Distribution

The FEM analysis of light distribution within the electrodes are performed by FEniCs module for Python by weak formulation of Helmholtz equation as shown in Eq. (2) in the main text. The Helmholtz equation is integrated with weak formulation as followed:

$$\int v\, \nabla^2 u dx + \int v k^2 u = 0$$

By integral by part, the above equation can be changed into weak formula as followed where left part is bilinear part.

$$-\int \nabla v \cdot \nabla u dx + \int v\, (\nabla u \cdot n) ds + \int k^2\, v u dx = 0$$

for the boundary condition for incident light, sommerfield irradiation condition was utilized as followed:

$$-\nabla u \cdot n = 2u_0 k i \exp\ (ikx)$$

For the interface between electrode and electrolyte, the Neumann boundary condition is utilized to express scattering layer which reflected light into the electrode as followed:

$$\nabla u \cdot n = 0$$

The geometry of calculated domain is meshed using Gmsh software and over 30,000 elements of triangular Lagrange type. In the FEniCs, complex number or complex function cannot by treated directly. Therefore, trial function, test function and wave numbers are all split into real part and imaginary part as followed:

$$u = u_r + u_i i$$
$$v = v_r + v_i i$$
$$k = k_r + k_i i$$

Then above weak formulation could be a bilinear function of u_r, u_i, v_r, v_i. The intensity of light is calculated from obtained data as followed:

$$I = u \cdot \bar{u} = u_r^2 + u_i^2$$

This calculated intensity is applied to intensity of light in the electron contiguity equation for analysis of J_{sc}.
If you want to obtain the source code, you can contact to corresponding author Dr. Cha (sicha@keri.re.kr).
